# 

**Published:** 1914-01-17

**Authors:** 


### CONTENTS.

PAGE

**Sir R. J. Collie on a State Medical Service** ... 409

**L'Affaire Lazarus-Barlow** ... ... ... ... 410

**Hospital and Institutional News** ... ... ... 411

Death of a London Hospital Chairman—A Personal Note—Illegal Resolutions by South Shields Guardians—Infirmary Officers and Maternity Benefit—A Woman Pioneer of Lunacy Reform—The New Heart Institution Opened—London Children and Motor-Ambulances—Truant Schools and Alternative Reforms—Once More the British Hospital for Mental Disorders—Street Collecting and New Institutions—A Coat of Arms for the Asylums Board—The Westminster Site—Annual Meetings in the Provinces—Medical Excuses—Lambeth Guardians and Accommodation for Babies—Death of a Well-known London Guardian—Fortune for the Cancer Hospital—Unnecessary Inquests and the Way Out—Non-Panel Doctor's Successful Protest—Hospital Contributions from Approved Societies—A Projected Private Hospital—The Police and the Hospitals—Unrest in the Asylums Board Ambulance Staff—Cottage Hospital "At Homes"—Attractions for Resident Medical Officers—Chelsea Hospital and the Palladium—The Insurance Act and the Drug Bills—This Week's Drug Market.

**The Grave Position at St. George's Hospital** 419

How Matters Stand.

Mr. West and St. George's Hospital.

**Report on Some Cottage Hospitals in Norfolk and Suffolk** ... ... ... ... ... ... 421

The Victoria Jubilee Hospital, Swaffham, Norfolk.

Thetford Cottage Hospital.

Mildenhall Cottage Hospital.

By Sir HENRY BURDETT, K.C.B., K.C.V.O.

[*See over.*]

### CONTENTS—(*continued*).

| | PAGE |
|---|---|
| **"Modern Methods" Series** ... ... ... | 417 |
| Gastro-Radiography—Metallic Meals. | |
| **Infantile Paralysis** ... ... ... ... ... ... | 418 |
| Vast Strides in Recent Researches. | |
| **Hospital Economics** ... ... ... ... ... ... | 423 |
| The Statistical Report of King Edward's Hospital Fund. | |
| **Hospital Statistics** ... ... ... ... ... ... | 424 |
| The Possibility of a Central Bureau. | |
| **National Insurance Act Developments** ... ... | 425 |
| Alterations in Administration of Maternity Benefit. | |
| **The New Institution for Diseases of the Heart** (illustrated) ... ... ... ... ... | 427 |
| A Tour of the New Buildings with the Secretary. | |
| **The Origin and Evolution of the 18th Century Hospital Movement** ... ... ... ... | 428 |
| **Round the World** ... ... ... ... ... ... | 430 |
| Fellow-workers and their Doings. | |
| **The New Poor-Law Orders** ... ... ... ... | 431 |
| The Duties, and a Policy for Medical Officers. | |
| **The Editor's Letter-Box** ... ... ... ... | 433 |
| The Crisis at St. George's Hospital. | |
| Where is the Limit to Christmas Festivities. | |
| Westminster Hospital. | |
| **The Profession of Medicine** ... ... ... | 434 |
| The National Medical Union. | |
| The Latest Developments of National Insurance. | |
| **The Institutional Worker** ... ... (Suppl.) | 1 |
| The Business Side of Christmas Festivities. | |
| **Our Bureau of Information** ... ... " | 2 |
| Optical Localiser. | |
| Employment and Training. | |
| The Sick and in Need. | |
| **Buildings Contemplated and in Progress** " | 3 |
| **The Book Market** ... ... ... " | 3 |

